# Mechanical and Microscopic Characteristics of Polyurethane-Based Pervious Pavement Composites

**DOI:** 10.3390/ma14164365

**Published:** 2021-08-04

**Authors:** Hongdong Cho, Hongsu Bae, Chanho Park, Hyeong Min Park, Seo-Eun Oh, Sang-Yeop Chung, Beomjoo Yang

**Affiliations:** 1 School of Civil Engineering, Chungbuk National University, 1 Chungdae-ro, Seowon-gu, Cheongju 28644, Korea; chohd84@hanmail.net (H.C.); bhs0038@chungbuk.ac.kr (H.B.); pch358925@naver.com (C.P.); ei.pothic@gmail.com (H.M.P.); 2 Department of Civil and Environmental Engineering, Sejong University, 209 Neungdong-ro, Gwangjin-gu, Seoul 05006, Korea; ohseoeun21418@sju.ac.kr

**Keywords:** pervious pavement, polyurethane binder, micro-CT analysis, mechanical properties, microscopic characteristics

## Abstract

Conventional pervious pavement materials (PPM) that consist of cement and aggregate materials are known for poor durability due to their brittle behavior. Thus, to enhance the durability, we fabricated polymeric PPMs from durable and abundant polyurethane (PU) and undertook mechanical and microscopic characterizations. PU-based PPM samples with varying aggregate sizes were produced and examined to test their compressive strength and water permeability. Furthermore, X-ray micro-computed tomography (micro-CT) was implemented to analyze the samples’ pore and tortuosity characteristics. Through the micro-CT analysis, the morphological characteristics of PPM’s internal structures were identified and quantitively analyzed the correlations between the pore size distribution, connectivity, and tortuosity within the samples. Finally, the microstructures derived from micro-CT were generated as a finite element model and also numerically determined the stress distribution generated inside.

## 1. Introduction

With regard to recent climate change technologies, the permeable function of pavement materials is gradually becoming more important to maintain material performance capabilities and improve urban environments [1,2]. The main benefit of permeable pavements is their ability to transport water through their structures, which prevents environmental damage caused by stormwater runoff or flooding [3]. Other environmental benefits of the permeable pavement include the ability to reduce urban heat island effects and prevent the penetration of harmful pollutants into the groundwater [4]. Construction pavement exposed to the outside air frequently undergoes expansion and contraction due to heating and cooling by weather, which can reduce the durability of these materials and lead to unpredictable accidents [5]. Abnormal climate change also quickly brings various defects, such as surface ripples, fractures, and cracks into pavement materials, ultimately increasing maintenance costs significantly [6].

In particular, the main surface wearing courses of concrete and asphalt roads undergo considerable temperature changes due to their high heat capacity and heat absorption rate [7]. Therefore, required repairs of construction pavement materials tend to increase significantly during the thawing season (January–March in Korea). In addition, pavement in which water permeation does not occur quickly is associated with many accidents caused by the freezing of roads in winter [8], as freezing on the road surface is generally difficult for drivers or pedestrians to recognize, and a low coefficient of friction increases the braking distance. Accordingly, several studies have been conducted in an effort to increase the permeability and durability of existing construction paving materials in order to improve urban sustainability and safety.

Li et al. [9] proposed a high-strength pervious type of concrete created with reactive powder concrete (RPC). It was experimentally proved that compressive strength and water permeability increase greatly when an amount of RPC is properly mixed with conventional pervious concrete. The authors also developed a precast design to increase the efficiency of the drainage system and prevent clogging. Pervious pavement blocks composed of bio-treated recycled aggregate were also investigated by Liu et al. [10]. A microbially induced calcium carbonate precipitation (MICP) process was introduced, and it was absorbed into the aggregate and dissolved in the mixing water. In both cases, the results showed an improvement in the mechanical properties of pervious pavement blocks, but the performance improvement was more pronounced when MICP was applied directly to water. In addition, various studies have been conducted in which byproduct aggregates or recycled aggregates are applied as components of permeable materials. Zaetang et al. [11] fabricated a specimen in which aggregates of pervious material were replaced with recycled aggregate and compared their mechanical properties. As a result, there was no significant effect on the tensile and compressive strength, but a significant improvement was observed in the compressive strength. It is presumed that the inherent adhesive properties between natural and recycled aggregates lead the experimental results, which is due to the difference in chemical composition among the aggregates [12]. It was noted that the amount of voids and the water permeability of the pervious specimens were greatly affected by the replacement ratio of byproduct aggregate [13].

When there are numerous voids in a material, the internal stress becomes concentrated around the voids, resulting in more rapid material destruction. Particularly, brittle materials undergo a remarkable reduction in their mechanical properties due to voids owing to their low tensile strength [14,15,16]. To address this issue, various studies of permeable pavement with an added polymer have been conducted. Huang et al. [17] conducted a laboratory experiment to improve the strength properties of pervious concrete through the incorporation of a latex polymer. Through their research, it was found that when natural sand and fibers are mixed into latex-incorporated pervious concrete, the water permeability is slightly reduced but the compressive and tensile strength levels are significantly improved. Giustozzi [18] applied four types of polymers (styrene-butadiene copolymer, vinyl-acetate homopolymer, ethylene vinyl-acetate copolymer, and styrene-butadiene copolymer) to permeable concrete and compared their effects on the formation of voids and the mechanical properties, finding that polymer materials were not very helpful when used to enhance the water permeability of pervious materials. However, they were confirmed to be capable of effectively improving the flexural strength, stiffness, and durability. Furthermore, non-destructive test (NDT) research to analyze the effects of internal voids in pavement materials on cracking and durability has been actively conducted in recent years [19,20,21,22,23].

## 2. Aim and Objectives

In the present study, a polyurethane (PU)-based pervious pavement material (PPM) was fabricated and examined by mechanical and microscopic characterizations. PU materials are widely used in the construction industry at present given their low cost and stable properties. In particular, the high tensile strength of PU is expected to lead to various performance improvements, including better durability and constructability [24,25,26]. PU-based PPM with varying aggregate sizes was produced and the compressive strength and permeability of each specimen were measured. The pore and tortuosity characteristics of the specimens were analyzed through X-ray micro-computed tomography (micro-CT). From the micro-CT analysis, the morphological characteristics of the internal structures of PPM were identified. In particular, the correlations between the pore size distribution, connectivity, and tortuosity within the specimen were quantitatively analyzed. The microstructures as determined by the micro-CT analysis were reconstructed as a finite element model, and the stress distribution generated inside was numerically ascertained.

## 3. Methods

### 3.1. Materials and Specimens

**Figure 1:**
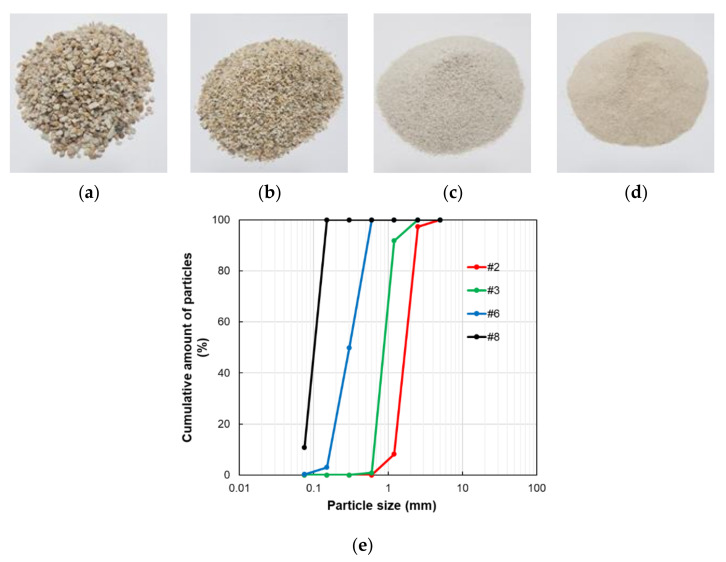
The appearances (**a**–**d**) and distribution curve (**e**) of the aggregates applied to the specimen.

In this study, the initial mix ratio of the polyurethane was determined by considering previous studies that focused on PPM. Metamorphic rock (quartzite) aggregates in four different size ranges were considered for the manufacturing of the test specimens. Based on Korean standards, aggregates #2, 3, 6, and 8 were applied, as listed in Table 1. The sizes of aggregates #2, 3, 6, and 8 were 40–65, 25–50, 5–40, and 2.5–10 mm, respectively. Figure 1 shows the four different types of aggregates adopted in this study. A smaller number in the name of the aggregate indicates a larger and coarser aggregate size. The chemical composition of utilized aggregate was analyzed through the X-ray fluorescence spectrometric analysis, as shown in Table 1.

The mix ratio of each specimen was based on the amount of aggregate #2 used in the specimen and the labels for the specimens were derived by the weight ratio of aggregate #2. For instance, specimen G33 signified that the weight of aggregate #2 out of the total weight of the specimen was 33%, with the weight ratio of the polyurethane binder set to 9%. Likewise, a total of 4 cases were designed and produced in this study to clarify the effect of mix composition, such as G47, G60, and G72 according to the amount of #2 aggregates. Figure 1a–d shows the appearances of each aggregate (#2, 3, 6 and 8) applied to the specimen. The distribution curve of the aggregates is also shown in Figure 1e [27]. The polyurethane used here consisted of a resin (HS-S100A, Hanseo Polymer Inc., Cheonan, Korea) and a hardener (HS-S100B©, Hanseo Polymer Inc., Cheonan, Korea) The weight ratio of the resin and hardener was 1:2.36. The ratio of resin and hardener of the polyurethane binder was determined by the manufacturer’s recommendation. In the present study, we intended to analyze the material mechanism of the pervious material according to the particle size of the aggregate with the B/A fixed. Experiments with different variables may result in more varied results, but it was judged that it might confuse the analysis of the phenomenon.

**Figure 2:**
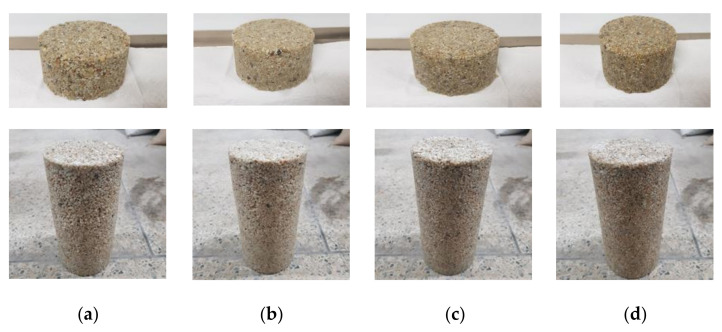
Fabricated test specimens for the (top) water permeability and (bottom) compressive strength tests: (**a**) G33, (**b**) G47, (**c**) G60, and (**d**) G72.

The specimens were manufactured via the following procedure: the coarse aggregates were combined depending on the mix ratio shown in Table 2 and blended for three minutes using a handheld mixer. In another container, the polyurethane resin and hardener were combined and blended for one minute. The blended polyurethane binder was poured into the container holding the blended coarse aggregates and mixed for another five minutes. The mixture was poured into a cylindrical concrete mold with a corresponding radius and height of 100 and 200 mm (*ϕ*100 × 200) and was then compacted. The cylindrical mold was demolded after being kept at room temperature for three days. Subsequently, the specimen was cured at room temperature for another seven days and was used for the compressive and water permeability tests. For the water permeability test, the test specimen was prepared by cutting the specimen into thirds horizontally. Figure 2 shows the appearance of the specimens fabricated for each formulation.

### 3.2. Characterizations

**Figure 3:**
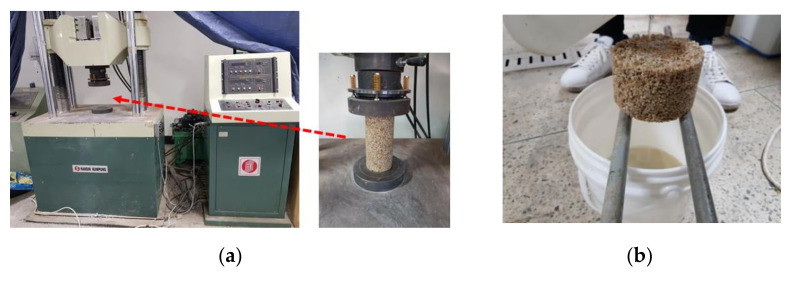
Experimental setup of (**a**) the compressive strength and (**b**) the water permeability test.

Figure 3 shows the test setup for the compression tests and the water permeability tests conducted as part of this study. The compression test was carried out following ASTM C 39 standards and was conducted using a universal material testing machine (UTM) with a loading rate of 1 mm/min [28]. Three specimens of each mixture were made and used to monitor the compressive behavior. The compressive strength was calculated by averaging the test results from the three test cases. In contrast, the water permeability test was conducted on pieces of a test specimen that had been cut horizontally into thirds and placed on two rods. Then, water was poured over top of each piece of the test specimen. Note that the water permeability test was not conducted in accordance with the standards but was conducted in consideration of field applicability. The field application thickness of the pervious pavement materials developed in this study is expected to be 20–30 cm. Therefore, only the assessment was made on whether water passed through a pervious material of height for one-third of the compressive strength specimen. The amount of water passing through the specimens was not quantitatively measured in this study.

**Figure 4:**
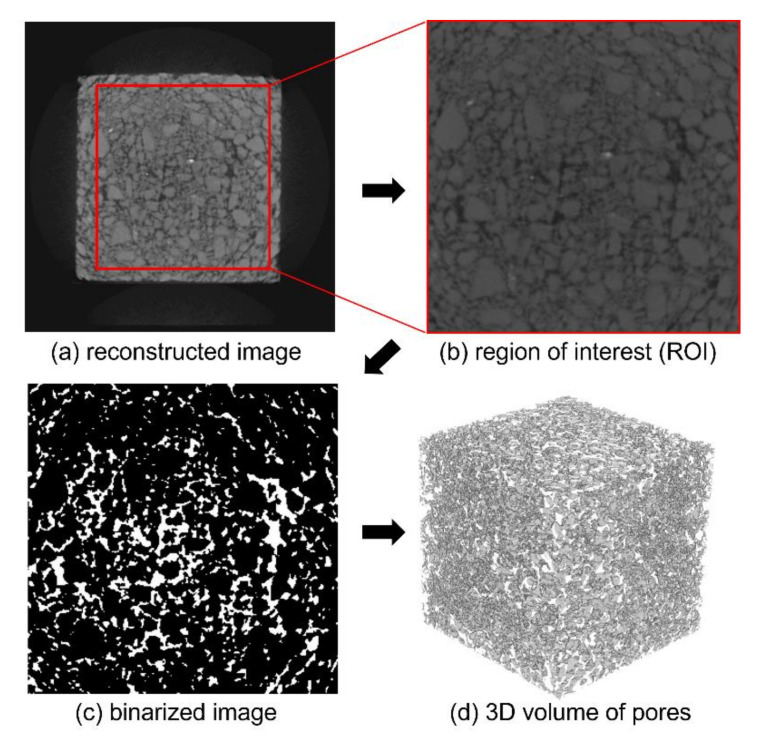
Micro-CT imaging process: (**a**) the original reconstructed image, (**b**) ROI image, (**c**) binary image, and (**d**) 3D volume image (Note: in (**c**,**d**), the white region represents the pores within the specimen).

In addition to the experimental approaches, micro-CT was also adopted to investigate the microstructural features of the specimens. Figure 4 shows the representative micro-CT imaging procedure of the G33 specimen. From the micro-CT measurement, the reconstructed image shown in Figure 4a was obtained. For the measurement, SkyScan1173 (Bruker, Billika, MA, USA) was used, and the measurement conditions were 130 kV and 61 mA. The cubic samples with 50 mm of edge length were used, and the reconstructed image is composed of 512 × 512 pixels with a pixel size of 146 μm, sufficient for the identification of pores within the produced specimens. For a more effective investigation, a region of interest (ROI) was selected from the original (reconstructed) image. The region of interest (ROI) image in Figure 4b is described as having 300 pixels along the edge with a pixel size identical to that in the original image. The reconstructed and the ROI images are 8-bit grayscale images represented by 256 values (0–255). From these grayscale images, the specific components of the target material can be segmented and utilized for a detailed analysis.

Given that the pore characteristics are the most critical when determining the properties of pervious materials, a binary image to describe the pore region was generated, as shown in Figure 4c. To segment the pore region from the original image, a proper threshold must be selected, and the modified Otsu method [29] was used here. In the binary image, white represents the pores within the specimen. A 3D volumetric image of the pores can then be obtained by the subsequent staking of a series of binary images, as shown in Figure 4d. The 3D volume of pores was used to investigate the quantitative pore characteristics of the specimens, in this case the porosity; this measure can also be utilized to examine the material properties using a numerical approach. The porosity was computed as the number of pore voxels compared to the 3D volume of the specimen, and only pores larger than 146 μm according to the image resolution were considered in the porosity analysis.

In addition to the porosity, which is an index by which to examine the quantitative pore characteristics, a parameter to evaluate the geometrical characteristics of the pore distribution is needed for a more detailed investigation of the specimens. For this purpose, tortuosity, an index that describes the curvature of a pore path, was adopted in this study. Tortuosity has been widely used to characterize the percolation characteristics of cement-based materials [30,31,32,33]. With the tortuosity investigation, the heterogeneity and connectivity of the pores within the porous specimens could be effectively described [34]. Tortuosity, denoted by τ, is defined as the ratio between the lengths of the actual path and the shortest path, as follows:

(1) $$\tau =L_{act}/L_{short}$$

In Equation (1), $L_{act}$ denotes the actual distance between two end points of a pore channel considering obstacles, while $L_{short}$ is the shortest length between the two end points.

**Figure 5:**
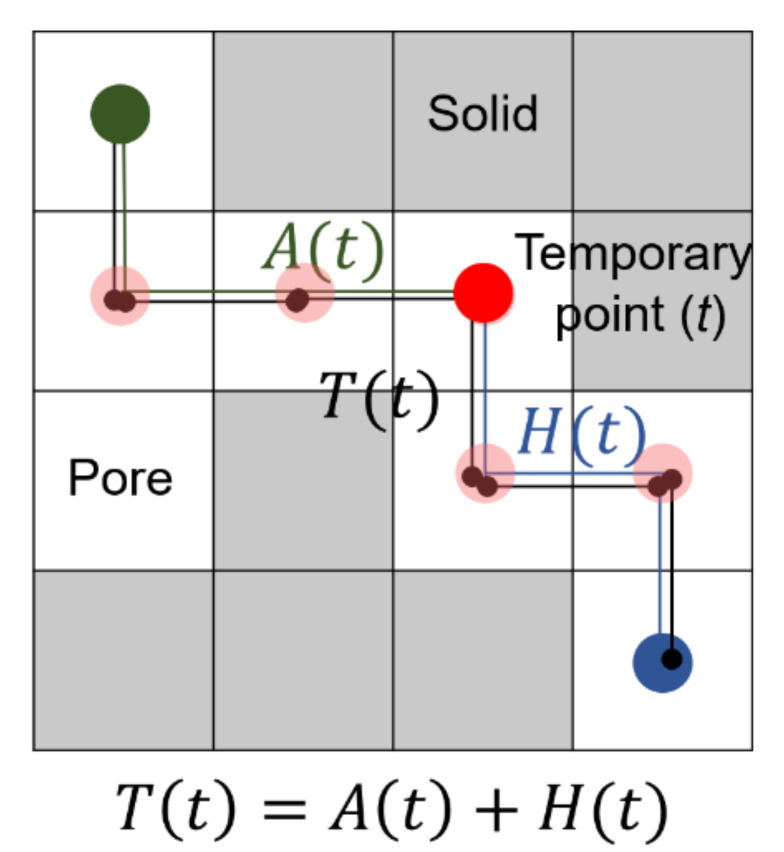
Schematic of the A-star algorithm (Note: *A*(*t*) is the actual distance between the starting point and a temporary point, and *H*(*t*) represents the heuristic path between the temporary and the end points at an iteration *t*. The total cost (*T*(*t*)) can be obtained by combining the actual and heuristic paths.).

To compute the actual distance ($L_{act}$), the A-star algorithm, a method capable of finding the minimum path between the selected starting and end points, was used [35]. Figure 5 shows a schematic of the A-star algorithm. This algorithm is composed of two parts: the actual path from the starting and a temporary point (*A*(*t*)), and the heuristic cost (*H*(*t*)) computed as the sum of vertical and horizontal routes between the temporary and the end points. The latter procedure is also called the Manhattan distance. The total cost (*T*(*t*)) was then calculated by adding *A*(*t*) and *H*(*t*). The computation of the total cost was repeated by moving the temporary point from the starting to the end points. The tortuosity values of the produced specimens were examined using the 3D volume image in each case, and the permeable characteristics of the specimens were discussed based on the tortuosity trends.

**Figure 6:**
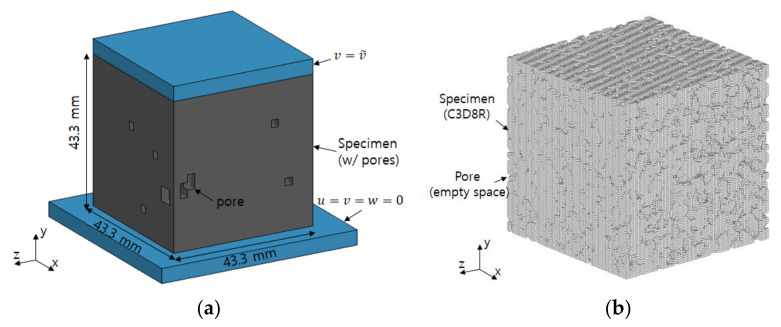
(**a**) FE-based simulation configuration and (**b**) the representative FE modeling (specimen G33).

With the obtained micro-CT data, the mechanical behavior of the specimens was also numerically investigated. Figure 6a shows a schematic of the analysis configuration considered in this study. The geometric configuration was extracted from the CT examination and converted to the ABAQUS format. The specimen was made of polyurethane-based PPM and contained internal and external pores throughout. The specimen had a height, width, and length of 43.3 mm, as indicated in this figure. The specimen was placed on the support and vertical displacement was applied for the compression test. Herein, the support and displacement plate were omitted in the finite element model by applying the displacement to the surface of the specimen directly.

Figure 6b presents the finite element model used for the analysis configuration. In this study, four types of specimens were considered, and this figure shows the typical case of specimen G33. This specimen was modeled using three-dimensional uniform hexahedral elements with a reduced integral point, with the pores left as empty spaces [36,37]. The numbers of elements used in specimens G33, G47, G60, and G72 were 801,947, 792,134, 749,337, and 701,929, respectively. Here, the number of elements for case 1, case 2, case 3, and case 4 were varied since the volume of pores in each specimen differed. It is assumed that each specimen responded to the compressive load in an elastoplastic manner [37].

## 4. Results and Discussion

### 4.1. Mechanical and Permeability Results

Table 3 presents the compression test results. While the compressive strengths for specimens G33, G47, and G60 were comparable to each other, the strength for specimen G72 was significantly low. The results from the water permeability tests in Table 3 showed that water drained through specimens G60 and G72, while specimens G33 and G47 absorbed water. It is thought that the size of aggregates and the combination of the aggregates affected the compressive strength and water permeability, as influenced by the connectivity between the internal pores in each specimen.

**Figure 7:**
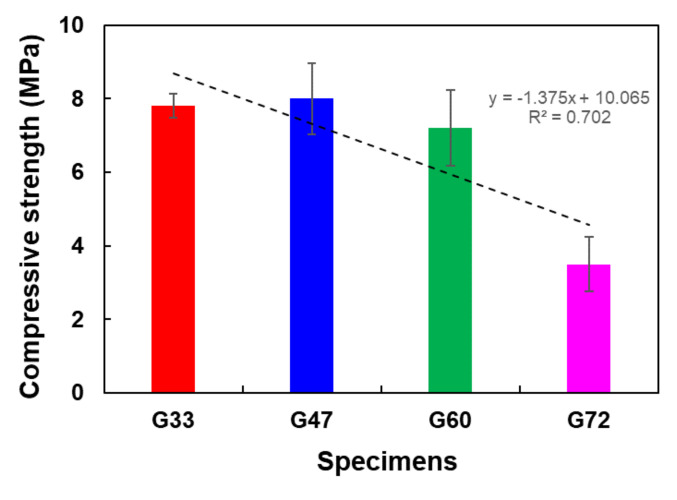
Compressive strength results of PPM specimens.

While concrete materials that are made with a coarse aggregate usually have low connectivity, which results in low strength, the compressive strength of the polyurethane-binder-based specimens here retained a high value despite the fact that the weight of aggregate #2 out of the total weight of the specimens varied from 33 to 60 wt.%. As the weight ratio of aggregate #2 increased to 72%, the compressive strength dramatically decreased. This trend remained although the pores in specimen G72 were filled with aggregate #8, which was the finest size considered in the study. Figure 7 shows the trends of compressive strength of specimen with respect to weight fraction of #2 aggregates. As the mixing amount of #2 aggregate increases, the compressive strength generally decreases, and the *R*^2^ value is approximately 0.7. A micro-CT examination and finite element method based numerical simulation were conducted to investigate this attribute in the following section.

According to Tennis et al. [38], the compressive strength of pervious concrete mixture is in the range of 3.5–28 MPa, and the typical value is approximately 17 MPa [38]. Higher compressive strength results have been reported when binders are prepared by mixing cement and polymer: Giustozzi [38] fabricated pervious specimens with a compressive strength of 50 MPa by applying cement and vinyl-acetate binder simultaneously. In addition, pervious materials with epoxy and polyurethane are reported to have compressive strengths of 6.0–14.1 and 5.6–6.9 MPa, respectively [39,40]. The compressive strength of 36 MPa was also recorded in the case of a pervious material using vinyl acetate ethylene and acrylic emulsion synthetic polymers as binder [40]. In summary, the compressive strength of about 7 MPa in the present experimental results is at an average level. Herein, the mechanism according to the internal properties of PPM was analyzed in more depth through micro-CT and numerical analyses.

### 4.2. Microstructural Characteristics

#### 4.2.1. Analysis of Pore Characteristics

**Figure 8:**
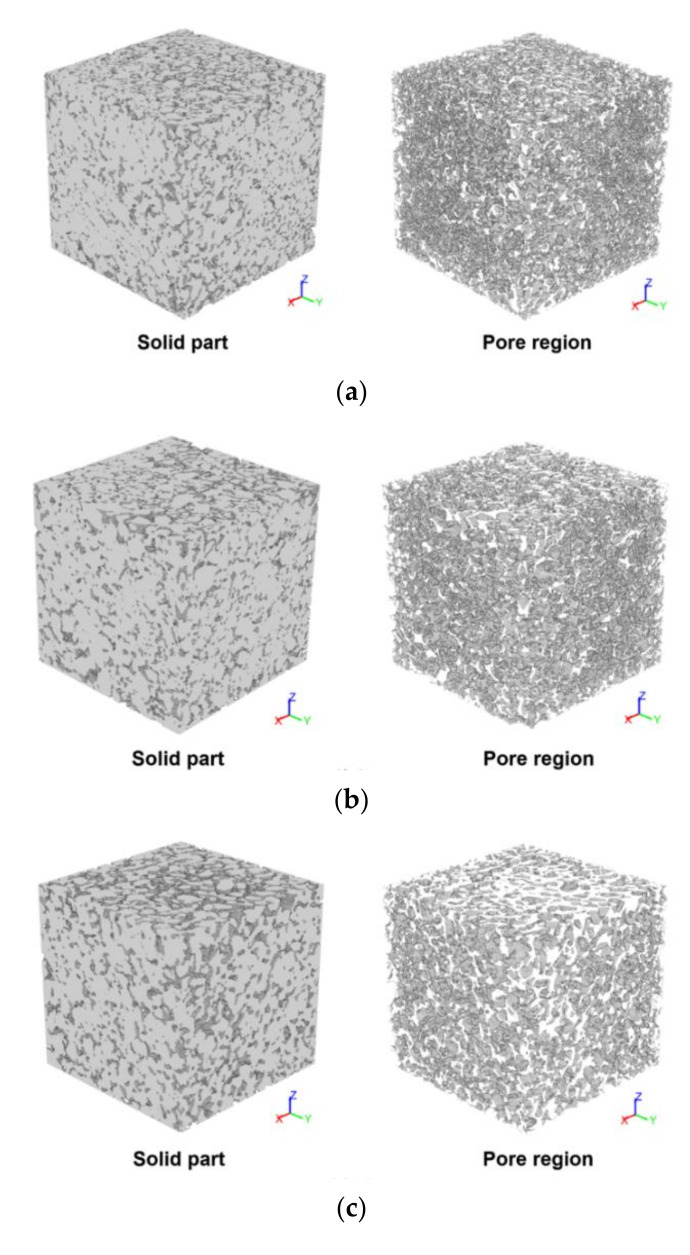

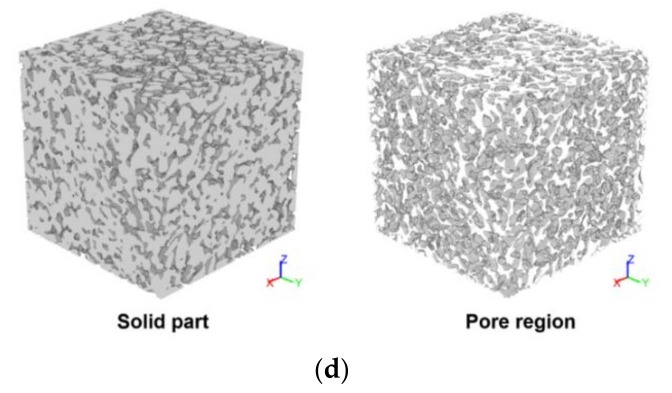
Segmented images of each specimen: (**a**) G33, (**b**) G47, (**c**) G60, and (**d**) G72 (note: in each figure, the solid part is on the left, and the image on the right represents the pore region of the specimen).

The microstructural characteristics of the specimens were investigated by examining micro-CT images of each sample. Figure 8 shows segmented images of each specimen, as obtained from micro-CT image processing. In this figure, the image on the left represents the solid part of the specimen, while the image on the right is the pore region of each sample. A solid mesh can be adopted for the finite element analysis to evaluate the mechanical behavior, as discussed in the following section. In addition, the 3D volume of the pores can be used to investigate the pore characteristics of the specimens.

From the pore images in Figure 8, it was noted that the pore structures of the G60 and G72 specimens tended to be coarser than those of the G33 and G47 specimens. To examine the quantitative pore characteristics, the porosity of the specimens was computed using the pore volume in Figure 8. Here, only pores larger than 146 μm were considered, and the measured porosity values of the G33, G47, G60 and G72 specimens were 19.55%, 20.55%, 24.81% and 29.50%, respectively. The qualitative and quantitative investigations both indicated that the specimens became more pervious as the specimen number increased. It was also found that the volume of pores in the materials varied up to 10% depending on the size of the aggregate, although the mix ratio of the polyurethane was held constant. In general, the pore structure of the material strongly affects the material properties, specifically the compressive strength and water permeability, and these differences in the porosity according to the aggregate size influenced the properties of the specimens produced in this study.

#### 4.2.2. Tortuosity Investigation

**Figure 9:**
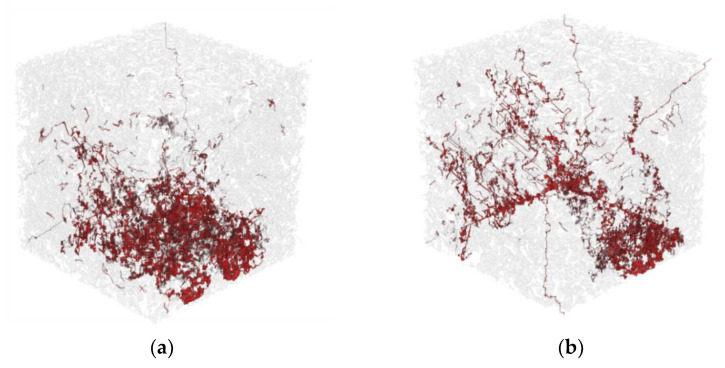

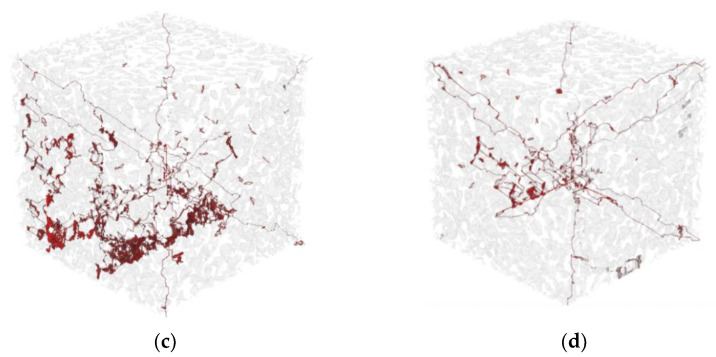
Pore paths for tortuosity in the specimens: (**a**) G33, (**b**) G47, (**c**) G60, and (**d**) G72 (note: the red regions represent the pore paths considered in the present study.).

For a more detailed analysis of the pore structure, the tortuosity, an index by which to examine the complexity of the pore path, was investigated for the target specimens. Figure 9 shows the percolation paths of each specimen, which can affect the tortuosity as they pass through the entire specimen. In this figure, the G33 specimen contains relatively few paths compared to the other cases, particularly the G60 and G72 specimens. Specimens with more paths can have a higher possibility in that water or fluid can flow, and this can be confirmed by quantitative measurements of, for instance, the tortuosity.

**Figure 10:**
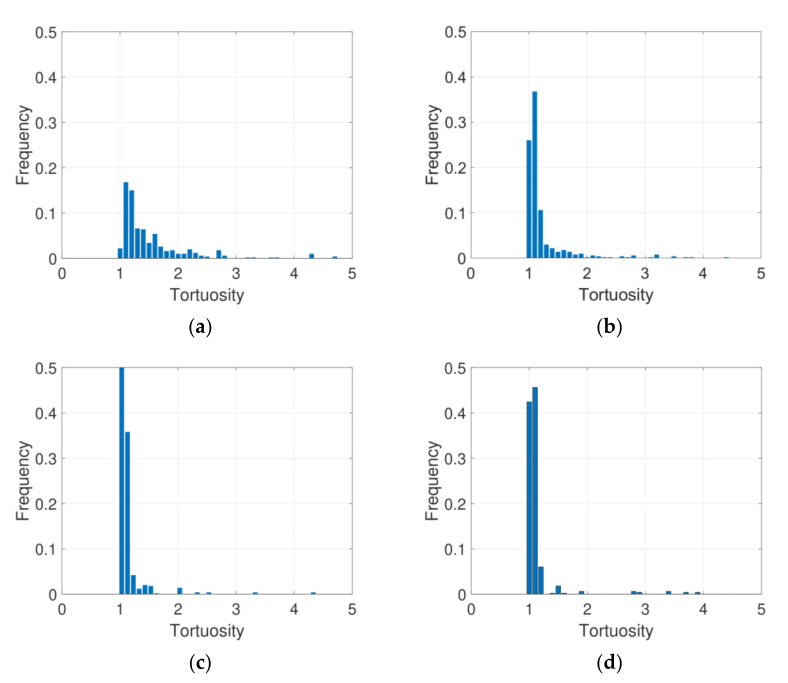
Tortuosity distribution of each specimen: (**a**) G33, (**b**) G47, (**c**) G60, and (**d**) G72.

Figure 10 shows the tortuosity distribution of the specimens considered here. In this figure, the *x*-axis denotes the tortuosity values as computed by the number of voxels, and the *y*-axis represents the frequency of each tortuosity value. Here, tortuosity is defined as the ratio between the actual path and the shortest distance, and this index can have a value larger than one. The tortuosity value increases as the specimen contains a more complex pore path, meaning that such a specimen can be considered as relatively less permeable. In the cases studied here, the G33 specimen tended to have the most dispersed tortuosity with the largest portion of the tortuosity exceeding a value of two; this indicates that the G33 specimen had the most complex and curved pore structure and was the least permeable among the specimens in this study.

Compared to the other cases, the tortuosity of the G60 and G72 specimens was mostly around one, which indicated that the specimens contained a wide and continuously connected pore network through which fluid could flow. These results indicate that the tortuosity is related to the porosity and relative pore size of the specimens, but its distribution trend can differ even in cases with a similar porosity range. Therefore, in addition to porosity, tortuosity can be used to describe the permeability-related characteristics of pervious materials.

### 4.3. Numerical Results

**Figure 11:**
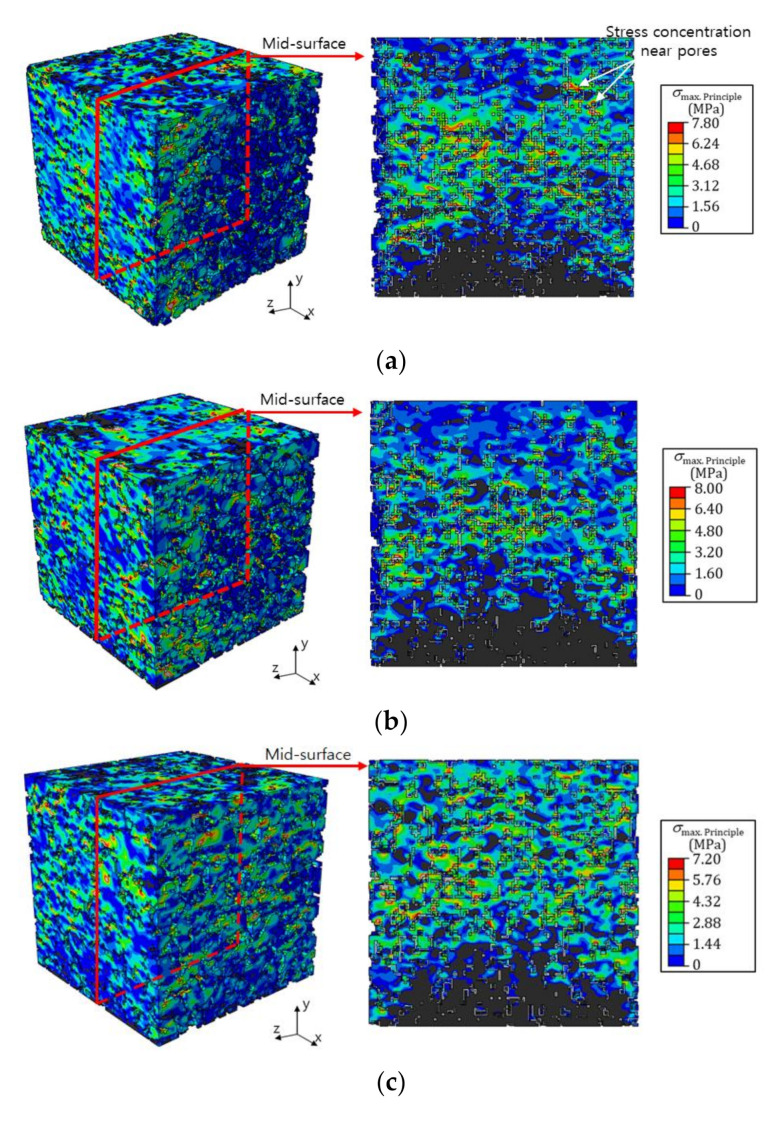

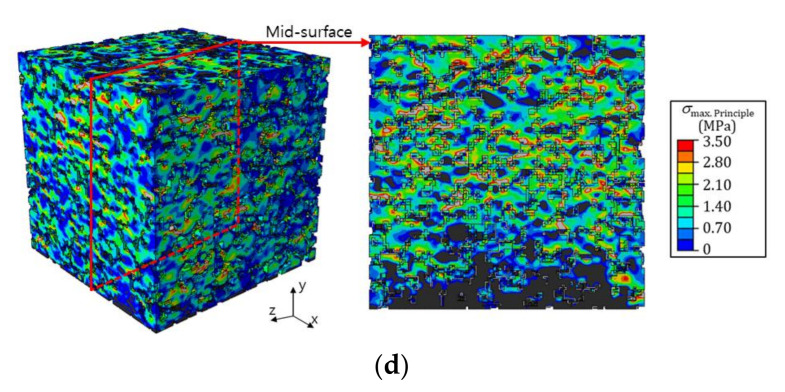
Stress distribution of PPM specimens under uniaxial compressive loading: (**a**) G33, (**b**) G45, (**c**) G60, and (**d**) G77.

In this study, the compressive behavior of PPM specimens was numerically simulated using a finite element model that was devised using geometric information extracted by a micro-CT examination. Through a series of experimental tests, the elastic modulus, compressive strength, and plastic strain for the pervious pavement composite were determined. With the modulus, compressive strength, and plastic strain, the elastoplastic constitutive relation for the composite material considered in this work was defined and applied to the FE model. Four types of specimens were considered, and each specimen included pores throughout its material. Figure 11 displays the stress distribution of the specimens under compression loading. Here, the PPM was assumed to act as an elastic material, and the maximum principal stress was applied as the stress indicator [41]. These figures are presented when the stress reached the compressive strength, as determined by the experiment.

As shown in the figures, stress developed through the entire specimen under compressive loading, and a large amount of compressive stress was established in the lower region of each specimen. The developed stress concentrated around the pores throughout each specimen and developed into large stress regions [42]. It is thought that the failure behavior may have initiated around the pores where these stress concentrations developed.

As presented in the micro-CT examination and in the finite element model, the volume and formation of pores in the specimens were varied and stress concentrations occurred around the pores. Specimen G70, which had the largest volume of pores in its material, was relatively vulnerable to compressive loading since stress was established and developed into a large stress concentration around the pores, which may have led to a failure of the material [43]. Meanwhile, specimens G33 and G47, which had relatively small volumes of pores in their materials, presented large compressive strength values. It should be noted that the volume of pores in the materials significantly influenced the compressive strength of the pervious concrete material [44].

**Figure 12:**
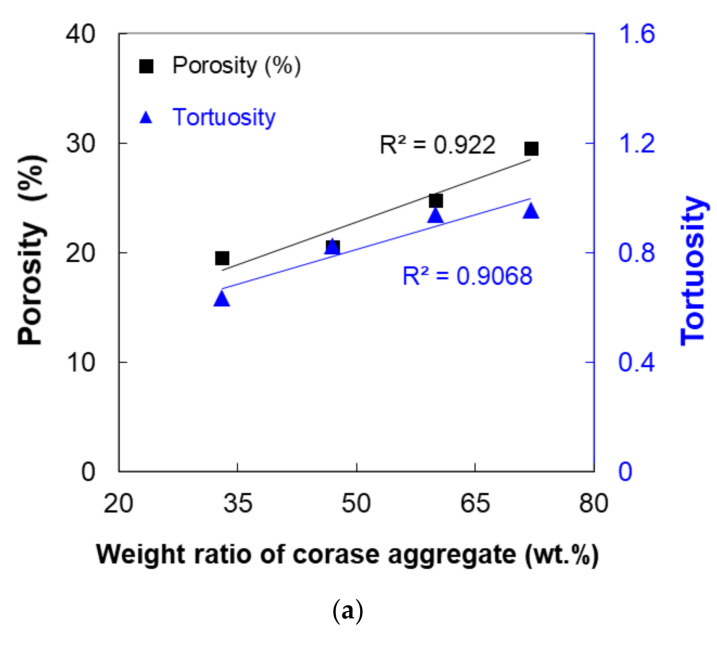

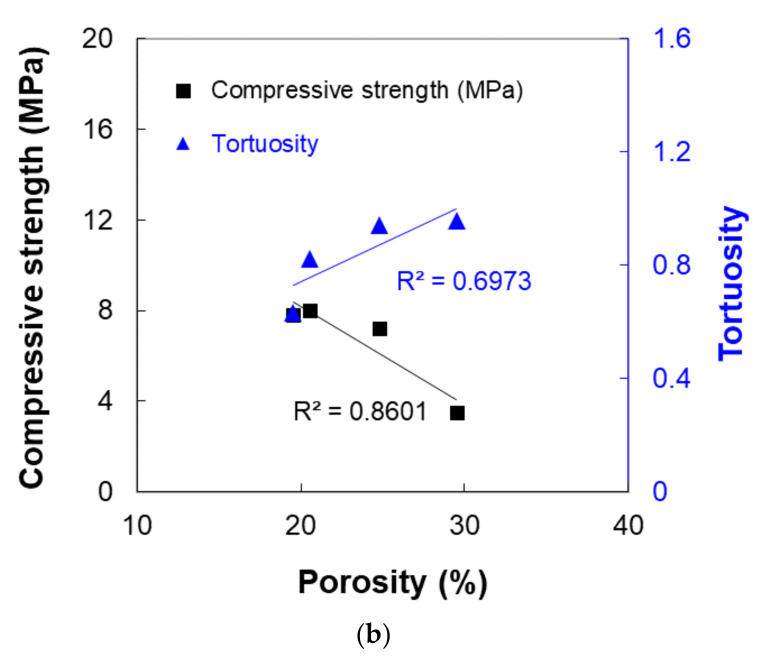
Correlation analysis between test variables: (**a**) effects of the weight ratio of #2 aggregate on the porosity and tortuosity, and (**b**) effects of the porosity on the compressive strength and tortuosity.

In addition, correlations between experimental variables were analyzed, as presented in Figure 12. It was found that the incorporated amount of aggregate at the maximum size (#2) was highly correlated with the porosity and tortuosity. Herein, the representative value of tortuosity (*τ*) was estimated by the following equation:

(2) $$\tau^{*}=\sqrt{\sum_{r=1}^{n}\left(\tau_{r}\cdot frequency_{r}\right)}$$

where the superscript * denotes the representative, the subscript *r* is the scale, and *n* indicates the overall value of the tortuosity.

The analysis showed that the aggregate size was highly correlated with the porosity and tortuosity. As the aggregate size was increased, both the porosity and tortuosity increased linearly (Figure 12a). In addition, the result in Figure 12b indicates that the porosity in the specimen is highly correlated with the compressive strength. As in the published literature [45,46,47], the material performance decreased as the porosity increased. The tortuosity value also increased as the porosity was increased, but there was no strong relationship (*R*^2^ = 0.69) compared to the compressive strength. This was judged as an experimental limitation due to the use of too few variables and to secure more certain experimental results regarding this aspect, additional research should be conducted in the near future. Comprehensive test results including compressive strength, porosity, and tortuosity shown in Figure 12 are also summarized in Table 4.

## 5. Concluding Remarks

The present study investigated the effects of the internal structures of PU-based pervious pavement materials on the compressive strength, pore size distribution, connectivity, and tortuosity. Experimental tests, microstructural analyses, and FE simulations of the specimens with different aggregate sizes were utilized, and the key findings thus obtained are summarized below.

(1) Compressive and permeability tests indicated that a combination of aggregate sizes had a significant effect on the pore path tortuosity.
(2) High contents of large-sized aggregate (#2) provoked an increase in the porosity and tortuosity, leading to high water permeability of these specimens.
(3) The specimen with a smaller aggregate size (2.5–10 mm) showed a dense internal structure, though this was not closely associated with the compressive strength development in this sample.
(4) The increased porosity contributed to a decrease in the compressive strength and an increase in the tortuosity, and the effects of the porosity on the tortuosity were insignificant compared to the compressive strength.

Porous materials such as pervious pavement are vulnerable to fatigue behavior, and thus dynamic testing considering time history is required for a more precise analysis. It may help to accurately characterize the transport load under laboratory conditions. The dynamic testing is beyond the scope of the present study; however, we plan to extend our work along this direction in the future.

## Figures and Tables

**Table 1:** The chemical composition of utilized aggregate (KS E 3076).

Chemical Composition	SiO_2_	Al_2_O_3_	TiO_2_	Fe_2_O_3_
Concentration (wt.%)	94.8	2.31	0.06	0.98

**Table 2:** Mix ratio of the polyurethane-based PPM.

Specimen	Weight Ratio of Aggregate (wt.%) ^1^				B/A ^2^
	#2	#3	#6	#8	
G33	0.33	0.33	0.33	0.00	0.09
G47	0.47	0.27	0.27	0.00	
G60	0.60	0.20	0.20	0.00	
G72	0.72	0.18	-	0.09	

^1^ Range of aggregate size: #2 = 2–5 mm, #3 = 1–2 mm, #6 = 0.2–0.4 mm, #8 = 0.1 mm or less. ^2^ Weight ratio of binder to aggregate (B/A). The weight ratio of the resin and hardener of PU is 1:2.36.

**Table 3:** Compressive strength and water permeability of the specimen.

	Specimens			
	G33	G47	G60	G72
Compressive strength (MPa)	7.8 ± 0.31	8.0 ± 0.97	7.2 ± 1.03	3.5 ± 0.74
Water permeability	X	X	O	O

**Table 4:** Comprehensive test results including compressive strength, porosity, and tortuosity.

	Specimens			
	G33	G47	G60	G72
Compressive strength (MPa)	7.8 ± 0.31	8.0 ± 0.97	7.2 ± 1.03	3.5 ± 0.74
Porosity (%)	19.55	20.55	24.81	29.5
Representative value of tortuosity	0.63	0.82	0.94	0.96

## Data Availability

Not applicable.
